# Supplementary material for: Genetically modified organisms and sustainable development goals: A survey of Taiwanese public opinion
Source: PLoS One. 2025 Jun 26;20(6):e0325790. doi: 10.1371/journal.pone.0325790 (PMC12200874; doi:10.1371/journal.pone.0325790)
Supplement: S1 File — Genetically modified organisms and sustainable development goals: a survey of Taiwanese public opinion. Genetically modified organisms and sustainable development questionnaire. (PDF) [file pone.0325790.s001.pdf]

Supplementary materials accompanying the manuscript

Genetically Modified Organisms and Sustainable Development Goals: A Survey of Taiwanese Public Opinion

Genetically Modified Organisms and Sustainable Development Questionnaire

Greetings!!

This is a questionnaire about Genetically Modified Organisms/Foods (GMOs) to find out what you understand, think and do about GMOs This questionnaire is anonymous and the information is for academic research purposes only, so please feel free to complete it! You can withdraw from this survey at any time during the process

Part I: Please describe your understanding of GMO (Genetically Modified Organism):
For each of the following questions, please select the one that best fits your idea

Question	Options				
1 Have you ever heard of the names “genetically modified organisms”, “genetically modified foods”, GMOs, etc? GMO”, ‘genetically modified food’, GMO, etc?	I've never heard of it	A little impressions	Have a clear impression	I've heard it all the time	I've always heard
2 How well do you think you know GMO?	Not at all	Understand a little bit	Forget it untie	Quite worthy untie	Strongly good untie

4 Question: Which of the following statements is true?	Options		
4-1 Genes are made of chromosomes	True	Not sure	False
4-2 Gene transfer does not occur in nature	True	Not sure	False
4-3 The more DNA an organism has, the higher it is	True	Not sure	False
4-4 Men and women have different numbers of chromosomes	True	Not sure	False
4-5 Consumption of GMO products may alter the genes of those who consume them change	True	Not sure	False

Part II: Ask your opinion on GMO (Genetically Modified Organism) Description: For each of the following questions, please choose the one that best fits your idea

**Question**	**Options**				
5GMO Violation of the laws of nature	Strongly Disagree	Disagree	Neutral Opinion	Agree	Strongly Agree
6GMO Violation of ethical principles	Strongly Disagree	Disagree	Neutral Opinion	Agree	Strongly Agree
7GMO Violation of religious beliefs	Strongly Disagree	Disagree	Neutral Opinion	Agree	Strongly Agree
8 I accept eggs with β-carotene	Strongly Disagree	Disagree	Neutral Opinion	Agree	Strongly Agree
9 I accept skim milk (removes the fat from the milk)	Strongly Disagree	Disagree	Neutral Opinion	Agree	Strongly Agree
10 I accept the proper use of preservatives to keep food fresh	Strongly Disagree	Disagree	Neutral Opinion	Agree	Strongly Agree
11GMO will bring good results for mankind	Strongly Disagree	Disagree	Neutral Opinion	Agree	Strongly Agree
12 Overall, I think GMO is worth encouraging	Strongly Disagree	Disagree	Neutral Opinion	Agree	Strongly Agree
13GMO has many unknown side effects	Strongly Disagree	Disagree	Neutral Opinion	Agree	Strongly Agree
14 GMO have Negative Health Effects on Adults	Strongly Disagree	Disagree	Neutral Opinion	Agree	Strongly Agree
15 GMO has negative impact on children's health	Strongly Disagree	Disagree	Neutral Opinion	Agree	Strongly Agree
16 GMO has negative impact on nature	Strongly Disagree	Disagree	Neutral Opinion	Agree	Strongly Agree
17 A bag of grain contains 01% GMO, I think the whole bag should be considered as GMO<br>I think the whole bag of grain should be considered as GMO	Strongly Disagree	Disagree	Neutral Opinion	Agree	Strongly Agree
18 GMO can increase global food production	Strongly Disagree	Disagree	Neutral Opinion	Agree	Strongly Agree
19 GMO can stabilize food prices	Strongly Disagree	Disagree	Neutral Opinion	Agree	Strongly Agree
20 GMO can reduce pesticide use	Strongly Disagree	Disagree	Neutral Opinion	Agree	Strongly Agree
21GMO can improve agriculture's ability to withstand climate change	Strongly Disagree	Disagree	Neutral Opinion	Agree	Strongly Agree

22 GMO can become an "edible vaccine" against diseases by implanting disease-resistant genes edible vaccine" to prevent disease	Strongly Disagree	Disagree	Neutral Opinion	Agree	Strongly Agree

**23 Question：Do you Agree with the following biotechnology?**	**Options**				
23-1 Genetically modified drugs	Strongly Disagree	Disagree	Neutral Opinion	Agree	Strongly Agree
23-2 Genetically modified plant	Strongly Disagree	Disagree	Neutral Opinion	Agree	Strongly Agree
23-3 Genetically modified animals	Strongly Disagree	Disagree	Neutral Opinion	Agree	Strongly Agree
23-4 Genetically modified plant food	Strongly Disagree	Disagree	Neutral Opinion	Agree	Strongly Agree
23-5 Genetically modified animal Food	Strongly Disagree	Disagree	Neutral Opinion	Agree	Strongly Agree

**Question**	**Options**				
24 GMO can be properly controlled by technology	Strongly Disagree	Disagree	Neutral Opinion	Agree	Strongly Agree
25 I have confidence in the government to manage GMO	Strongly Disagree	Disagree	Neutral Opinion	Agree	Strongly Agree
26 GMO technology will evolve beyond human control	Strongly Disagre	Disagree	Neutral Opinion	Agree	Strongly Agree

	e				
27 Public should be involved in GMO management	Strongly Disagree	Disagree	Neutral Opinion	Agree	Strongly Agree
28 I Agree with the education department's “no GMOs for school lunches” rule GMOs” in the education sector	Strongly Disagree	Disagree	Neutral Opinion	Agree	Strongly Agree
29 I Agree with the concept of “man's destiny is better than God's”	Strongly Disagree	Disagree	Neutral Opinion	Agree	Strongly Agree
30 Man is born with the right to govern nature	Strongly Disagree	Disagree	Neutral Opinion	Agree	Strongly Agree
31 Man has destroyed the environment to the point of near collapse	Strongly Disagree	Disagree	Neutral Opinion	Agree	Strongly Agree

Part III: Please describe your behavior in relation to GMO (Genetically Modified Organism): For each of the following questions, please select the one that best fits your actual behavior

Question	Options				
33 What is the likelihood that you will be exposed to GMO (food, clothing, housing and other lifestyle behaviors) in your life?	Not at all.	Not normally.	Common	Usually	Always
34 What is the likelihood of you eating GMO in your life?	Not at all.	Not normally.	Common	Usually	Always
35 Have you ever eaten GMO food?	Confirmed no.	I don't think so.	Neutral Opinion	I think so.	Confirmation

Question	Options
36 How much more would you be willing to pay for non-GM foods compared to GM foods?	□ 0%<br>□ 0~10%<br>□ 11~20%<br>□ 21~30%<br>□ 31~40%<br>□ 41~50%<br>□ 51~60%<br>□ 61~70%<br>□ 71~80%<br>□ 81~90%<br>□ 91~100%
37 How much more are you willing to pay for organic food compared to regular food?	□ 0%<br>□ 0~10%<br>□ 11~20%<br>□ 21~30%<br>□ 31~40%<br>□ 41~50%<br>□ 51~60%<br>□ 61~70%<br>□ 71~80%<br>□ 81~90%<br>□ 91~100%

Part IV: Ask your opinion on the relationship between Genetically Modified Organisms (GMOs) and Sustainable Development Goals (SDGs)

Instructions: For each of the following descriptions, please choose the one that best suits your idea

**Question**	**Options**				
38 Have you seen the following images?<br>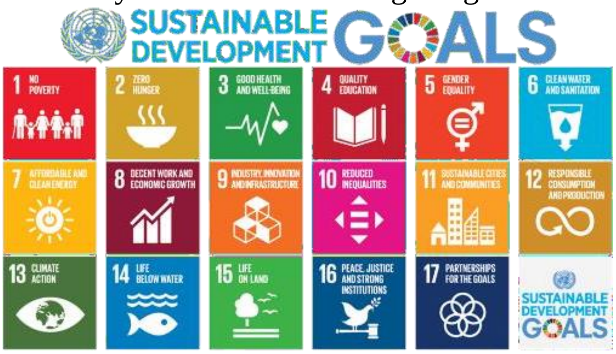 	True	False	Not sure		
39 Do you understand the SDGs (Sustainable Development Goals) advocated by the United Nations?	Not at all	Understand a little bit	Forget it untie	Quite worthy untie	Strongly good untie

**Question：40 What do you think is the degree of correlation between "genetically modified organisms" (GMOs) and individual "sustainable development goals" (SDGs)? Explanations: (1) If you think GMOs are Strongly helpful in promoting a particular SDG, then choose Strongly positive [+2]; (2) If you think GMOs are somewhat helpful in promoting a particular SDG, then choose Positive [+1]; (3) If you think GMOs are somewhat detrimental to achieving a particular SDG, then choose Negative [-1]; (4) If you think GMOs are Strongly detrimental to achieving a particular SDG, then choose Strongly Negative [-2]; (5) If you think GMOs are Strongly detrimental to achieving a particular SDG, then choose Strongly Negative [-4]; and (6) If you think GMOs are Strongly detrimental to achieving a particular SDG, then choose Strongly Negative [-5] (4) If you think the GMO is Strongly unfavorable to the achievement of an SDG, then choose Strongly Negative [-2]; (5) If you think the GMO is related to the achievement of a**<br>**(5) If you think that GMO is not at all related to the achievement of a certain SDG, then choose Normal [0]**	**Options**				
SDG 1: No Poverty	Strongly negative (-2)	Negative (-1)	Common (0)	Positive(+1)	Strongly positive(+2)
SDG 2: Zero Hunger	Strongly negative (-2)	Negative (-1)	Common (0)	Positive (+1)	Strongly positive (+2)
SDG 3: Good Health and Well-Being	Strongly negative (-2)	Negative	Common	Positive (+1)	Strongly positive

		(-1)	(0)		(+2)
SDG 4: Quality Education	Strongly negative (-2)	Negativ e(-1)	Commo n (0)	Positive (+1)	Strongly positive (+2)
SDG 5: Gender Equality	Strongly negative (-2)	Negativ e (-1)	Commo n (0)	Positive (+1)	Strongly positive (+2)
SDG 6: Clean Water and Sanitation	Strongly negative (-2)	Negativ e(-1)	Commo n (0)	Positive (+1)	Strongly positive (+2)
SDG 7: Affordable and Clean Energy	Strongly negative (-2)	Negativ e (-1)	Commo n (0)	Positive (+1)	Strongly positive (+2)
SDG 8 : Decent Work and Economic Growth	Strongly negative (-2)	Negativ e (-1)	Commo n (0)	Positive (+1)	Strongly positive (+2)
SDG 9 : Industry, Innovation and Infrastructure	Strongly negative (-2)	Negativ e (-1)	Commo n (0)	Positive (+1)	Strongly positive (+2)
SDG 10 : Reduced Inequalities	Strongly negative (-2)	Negativ e (-1)	Commo n (0)	Positive (+1)	Strongly positive (+2)
SDG 11 : Sustainable Cities and Communities	Strongly negative (-2)	Negativ e (-1)	Commo n (0)	Positive (+1)	Strongly positive (+2)
SDG 12 : Responsible Consumption and Production	Strongly negative (-2)	Negativ e (-1)	Commo n (0)	Positive (+1)	Strongly positive (+2)
SDG 13 : Climate Action	Strongly negative (-2)	Negativ e (-1)	Commo n (0)	Positive (+1)	Strongly positive (+2)
SDG 14 : Life Below Water	Strongly negative (-2)	Negativ e (-1)	Commo n (0)	Positive (+1)	Strongly positive (+2)
SDG 15 : Life on Land	Strongly negative	Negativ e	Commo n	Positive (+1)	Strongly positive

	(-2)	(-1)	(0)		(+2)
SDG 16 : Peace, Justice and Strong Institutions	Strongly negative (-2)	Negativ e (-1)	Commo n (0)	Positive (+1)	Strongly positive (+2)
SDG 17 : Partnerships for the Goals	Strongly negative (-2)	Negativ e (-1)	Commo n (0)	Positive (+1)	Strongly positive (+2)

Part V: Personal Informati

**Question**	**Options**	
41. What is your gender?	female	male
42. What is the year of your birth?		
43. What is your level of education?	☐ Junior college student<br>☐ Junior college graduate<br>☐ University student<br>☐ University graduate<br>☐ Graduate school student<br>☐ Graduate school graduate<br>☐ Other	

44. Which of the following is close to your professional field?	☐ Science<br>☐ Humanities and Social Science<br>☐ Other
